# Adjuvant electrochemotherapy in veterinary patients: a model for the planning of future therapies in humans

**DOI:** 10.1186/1756-9966-28-114

**Published:** 2009-08-14

**Authors:** Enrico P Spugnini, Gennaro Citro, Alfonso Baldi

**Affiliations:** 1 S.A.F.U. Department, Regina Elena Cancer Institute, Rome, Italy; 2 Department of Biochemistry, section of Pathology, Second University of Naples, Naples, Italy

## Abstract

The treatment of soft tissue tumors needs the coordinated adoption of surgery with radiation therapy and eventually, chemotherapy. The radiation therapy (delivered with a linear accelerator) can be preoperative, intraoperative, or postoperative. In selected patients adjuvant brachytherapy can be adopted. The goal of these associations is to achieve tumor control while maximally preserving the normal tissues from side effects. Unfortunately, the occurrence of local and distant complications is still elevated. Electrochemotherapy is a novel technique that combines the administration of anticancer agents to the application of permeabilizing pulses in order to increase the uptake of antitumor molecules. While its use in humans is still confined to the treatment of cutaneous neoplasms or the palliation of skin tumor metastases, in veterinary oncology this approach is rapidly becoming a primary treatment. This review summarizes the recent progresses in preclinical oncology and their possible transfer to humans.

## Introduction

Achieving local tumor control in cancer patients is one of the primary tasks of oncologists and is frequently cause of serious concerns. In fact, lack of awareness, inadequate screenings and the sudden onset of rapidly growing neoplasms often do not allow to eradicate cancer by using surgery alone. Therefore, due to the advanced stage at the time of diagnosis, cancer is preferentially treated with multi-modality therapies. In humans, these combinations have been tested through multi-institutional phase II and III trials and usually consist of the association of surgery and radiation therapy (either brachytherapy or radiation beam) [1-6]. Chemotherapy is usually confined to an adjuvant role for those cancers with high tendency to metastasize (i.e. high grade sarcoma or breast cancer) or is perfusionally administered in combination with hyperthermia for advanced disease [7-10]. However, the high costs of these treatments as well as the side effects of these procedures limit their widespread application [1,10,11]. Another crucial point when evaluating local therapies for advanced neoplasms is the biological cost paid by the patients. Sometimes the complications of aggressive surgery and radiation therapy may result in a poor quality of life. The most commonly reported side effects of radiation therapy are: 1) gradual side-effects, usually dose-dependent (local fibrosis, necrosis, nerve damage etc.) and 2) the so called "statistically demonstrable side effects", also known as "radiation induced tumors" [2,3].

The risk of side effects is particularly high when dealing with aggressive malignant neoplasms (Grade III with high mitotic rate). However, in case of large neoplasms that involve deep underlying structures, preoperative radiation therapy might be chosen in the attempt to shrink the tumor volume and to reduce the satellite infiltrations [5]. Unfortunately the rate of local wound complication associated with aggressive surgical management and radiation therapy is still elevated [6]. The incidence of these side effects cannot be reduced since several publications pointed out a trend toward increased disease free interval and survival in patients receiving multimodality treatments [7,9,10].

### Electrochemotherapy

A new cancer treatment that can achieve high rates of remission without the associated problems of high financial and biological cost of previous procedures has been explored over the past 15 years and called electrochemotherapy (ECT). It combines the administration of chemotherapy drugs with the application of permeabilizing pulses having appropriate waveform in order to enhance the captation of antitumor molecules by tumor cells.

Before its clinical adoption, *in vitro *studies showed that the application of high voltage, exponentially-decaying electric pulses to cells in suspension could induce "pores" in the cell membrane, thus resulting in cross-membrane flow of material or even in cell fusion if the cells were closely located [12-14]. Later, researchers discovered that electroporation could be instrumental to increase the delivery of drugs and plasmids through the cytoplasmic membrane by exposing animal cells in culture and plant protoplasts to adequate electric pulses [12-15]. In a second time, electroporation was used to improve the *in vitro *cytotoxicity of specific anticancer agents [16,17].

The first and still most actively used chemotherapy agent in ECT has been bleomycin. This drug can enter the cell membrane only through specific protein receptors, since its lipophobic nature prevents the simple diffusion, therefore resulting in slow and extremely limited uptake under normal conditions [16]. The complex formed by bleomycin and the membrane receptor is transferred within the cytosol through endocytotic vesicles. In the nucleus bleomycin rapidly causes DNA fragmentation, that is similar to that induced by radiation [16,17]. The high toxicity of bleomycin when it reaches the intracellular environment is limited by its impaired diffusion (less than 0.1% reaches its target in cultured cells) through the cytoplasmic membrane [16,17]. For these reasons, despite its therapeutic potential, the use of bleomycin has been limited in the clinical experience, until it has been shown that its cytotoxicity could be significally enhanced by electroporation, leading to a revival of this drug [17-22]. Another drug whose uptake can be increased by this mechanism is cisplatin (CDDP), however its captation is less influenced by the concurrent application of electric pulses, consequentially this agent has been less extensively investigated [23].

Several electroporation protocols have been adopted, mostly involving sequences of repeated decaying or square single pulses until the desired number of permeabilizing electric stimulations was reached [12-18]. More recently, a novel protocol involving the adoption of bursts of biphasic pulses with selectable period of repetition has been successfully used both in veterinary patients as well as in humans [19,24-31]. This schedule offers advantages in decreasing the morbidity of the treated animals and humans as well as improving the clinical outcome [19,24-32].

The exact mechanism of this therapy at the membrane level is not yet well understood, however recently consistent membrane changes have been shown by electron microscopy, following the exposure to electric pulses of melanoma tumors transplanted in mice [33]. Specifically, the freeze-fracturing analysis "evidenced defects in the dynamic assembly of lipids and proteins in both models, which ended up with the formation of "areas with rough structure" and intensive clustering of intramembrane proteins" [33]. These changes are suggestive of lipid and protein alterations, of altered protein cohesion and, perhaps. polarity, as well as of changes in lipid orientation within the cell membranes. Finally, the intercellular flow of microvescicle among cancer cells was disrupted following the destruction of these organelles by the electric pulses, probably inducing an impairment of cytokines and intercellular signal pathway.

### Results obtained in pets with spontaneously occurring neoplasms

Differently from other cancer investigations, electrochemotherapy has frequently conducted at the same time studies in rodents and in companion animals.

The first *in vivo *study involved the use of ECT as a rescue protocol in cats with recurring soft tissue sarcoma after adjuvant radiation therapy [18]. In that trial, cats were randomized to receive bleomycin ± the implant of 30 × 10^6 ^CHO cells (secreting interleukin 2) followed by the application of square pulses. The study was completed by a small cohort of untreated cats that acted as control. The authors described only one partial response however, they claimed a prolonged survival in 12 cats receiving ECT versus 11 untreated controls. This minimal response rate could be partially due to the previous treatments that led to the development of chemoresistance. In fact, it is known that radioresistant neoplasms have increased DNA repair which is one of the described mechanisms of resistance to bleomycin as well, at least in cell lines [15].

After this preliminary investigation, two phase I/II studies were conducted in companion animals; in the first a cohort of dogs and cats were treated with intralesional cisplatin coupled with square electric pulses [23] while in the second they received intralesional bleomycin driven by trains of biphasic pulses [19]. The overall response rate of this second investigation was 80% with a 40% of long lasting remissions. This study evidenced that among the treated neoplasms, canine hemangiopericytomas were particularly responsive to this approach. This work evidenced two problems of ECT: the need of specifically tailored electrodes for the therapy of soft tissue neoplasms and the major obstacle to a smooth permeabilization represented by the high content of connective tissue within solid tumors [24]. Currently, ECT is preferentially adopted as single modality only for tumors very susceptible to electroporation such as melanomas and perianal adenomas [34-36] or relatively small in size and easily accessible like sun-induced nasal carcinomas [29]. In selected patients with cutaneous epitheliotropic and non-epitheliotropic lymphoma this therapy can lead to successful palliation or even extended local control and, consequently, survival [37].

After the development of novel electrodes [25], several phase II studies were conducted in our Institution to evaluate the potential of ECT as adjuvant treatment after surgical cytoreduction of bulky tumors mimicking the protocols of intraoperative radiation therapy [38].

A preclinical study involving cats with soft tissue sarcomas, evaluated the potentials of intraoperative and postoperative ECT [26]. Cats were randomized to the following groups: surgery single modality, surgery plus intraoperative ECT and surgery plus postoperative ECT. The study underlined the significant advantage offered by adjuvant ECT in terms of local control and overall survival compared to surgery alone. Time to recurrence was 12 and 19 months for the intraoperative and postoperative cohorts respectively, while the tumors treated with surgery alone recurred within an average of 4 months. The results compare favorably with those of the current veterinary oncology therapies. The difference in local control times can be ascribed to the decision to enroll in the intraoperative group cats with rapidly growing neoplasms, leading to greater electroporation fields. One critical advantage of this technique is the possibility to repeat the treatment in selected patients experiencing local recurrence without the side effects of re-irradiated tissues [26].

A similar study in 22 dogs with soft tissue sarcomas, preferentially treated with a postoperative protocol, yielded a median time to recurrence of 730 days with a 95% response rate, and again hemangiopericytoma showed to be extremely sensitive to ECT, data confirmed by results obtained in cats as well [27,39]. The side effects of veterinary patients treated with adjuvant ECT were confined to local inflammation and occasional wound dehiscence [26,27].

Concurrently, adjuvant ECT has been tested in a cohort of 28 dogs with mast cell sarcomas, resulting in a response rate of 85% and a mean time to recurrence of 52.7 ± 6.5 months, moreover the authors reported that at the time of writing the median time to recurrence was not reached yet, since 24 of the patients were still disease free [28]. Two patients experiencing marginal recurrence were successfully treated with a minor surgery combined with a single application of electrochemotherapy [28]. The use of ECT is not strictly limited to superficial neoplasms: there is also some evidence that trains of biphasic pulses can improve the local control of incompletely excised deep perianal tumors, with preservation of organ function [35,36,40]. Caution should be exerted when adopting ECT as a rescue in patients that failed radiation therapy. A case report describes a severe radiation recall in a cat treated with adjuvant radiation therapy for a recurring fibrosarcoma [41]. Interestingly, this cat has been locally treated with cisplatin rather than bleomycin and perhaps the reaction has been triggered by the local administration of a platinum compound, since it is among the drugs linked with this type of complication [42]. Table 1 summarizes the results obtained in companion animals carrying spontaneous tumors that have been so far treated with electrochemotherapy.

**Table 1:** Summary of treatment outcome for cancer pets receiving electrochemotherapy

Drug	Species	N° of patients	Tumor Type	Response	References
B	Cats	12	STS	SD 7 months	18
B	Dogs and cats	16	Various	80% CR+PR	19
CDDP	Dogs and cats	7	Various	84% CR	23
B	Dogs and cats	5	Various	CR > 24 mo	25
B	Cats	58	STS	Median time to recurrence: 12–19 mo	26
B	Dogs	22	STS	Median time to recurrence 24 mo	27
B	Dogs	28	MCT	82%CR > 22 mo	28
B	Cats	9	SCC	78%CR> 3 mo	29
B	Cats	1	Ganglioneuroblastoma	CR > 15 mo	30
B	Dogs	10	Melanoma	70% CR> 6 mo	34
CDDP	Dogs	1	Anal Melanoma	PR 3 mo	35
B	Dogs	12	Perianal tumors	83% CR	36
B	Dogs and Cats	6	Lymphoma	100% CR 1 wk to 36 mo	37
B	Cats	1	HPC	CR> 12 mo	39
CDDP	Dogs	1	Anal sac carcinoma	CR> 18 mo	40
CDDP	Cats	1	Fibrosarcoma	CR 3 mo	41
Mitoxantrone	Dogs	1	Metastatic carcinoma	CR > 6 mo	43

B Bleomycin; CDDP Cisplatin; CR Complete Remission; HPC Hemangiopericytoma; MCT Mast Cell Tumors; PR Partial Remission; SCC Squamous Cell Carcinoma; SD Stable Disease; STS Soft Tissue Sarcoma.

## Conclusion

ECT has proven to be a safe and efficacious therapy for the local control of soft tissue neoplasms in companion animals, and its effectiveness is especially strengthened when used in an adjuvant fashion through the generation of trains of biphasic pulses [15,21-37,39-41,43]. ECT is currently being assayed for different spontaneous tumors in companion animals showing promising results and identifying patterns of response and clinical [25-27] as well as histopathological prognostic factors [31]. Further studies are currently ongoing to evaluate new drugs and delivery systems to improve the responses obtained so far, in particular mitoxantrone is a drug that is showing considerable promise [43], also in view of its future applications to human patients.

## Competing interests

The authors declare that they have no competing interests.

## Authors' contributions

EPS and AB equally contributed to this work, GC supervised the other contributors and critically revised the manuscript.
